# The Trajectory from Mood to Obesity

**DOI:** 10.1007/s13679-017-0291-6

**Published:** 2017-12-07

**Authors:** Judith Wurtman, Richard Wurtman

**Affiliations:** 1 Department of Brain and Cognitive Sciences, Massachusetts Institute of Technology, Cambridge, MA 02139 USA; 2 300 Boylston St., Unit 1205, Boston, MA 02116 USA

**Keywords:** Depression, Anxiety, Obesity, PTSD, Serotonin, Weight gain, Dietary carbohydrates

## Abstract

**Purpose of Review:**

To describe and explain the relationships between mood disturbances and the development of obesity.

**Recent Findings:**

That depression, anxiety, PTSD, or severe stresses can promote obesity as a side-effect of the drugs used to treat them, or through “carbohydrate craving” to enhance brain serotonin synthesis and alleviate dysphoria by consuming foods that are rich in both carbohydrates and fats. That seasonal affective disorder and severe PMS can independently cause patients to overconsume foods rich in both carbohydrates and fats.

**Summary:**

The obesity caused by drugs or mood disorders associated with “carbohydrate craving” leading to excess calorie intake can be suppressed by dietary measures.

## Introduction

Halting the continuing rise in obesity among adult Americans, may depend, in part, on being able to identify behavioral disorders associated with overeating and weight gain and intervening before this leads to obesity [1]. Recent epidemiological studies have shown that clinical depression in adolescence or early adulthood frequently precedes the development of adult obesity [2]. Moreover, other depression-related mood disturbances, for example severe premenstrual syndrome (late luteal phase dysphoric disorder; LLPDD), chronic anxiety with binge eating [3], and post-traumatic stress syndromes [PTSD] [4•], can also be associated with substantial weight gain, even occurring some years after the events which incited the PTSD [4•].

The association of mood disorders with the propensity to become obese might, for some patients, reflect a side effect of antidepressants or other behaviorally-active drugs used for treatment [1, 5, 6]. Some such drugs affect eating behavior by diminishing satiety and by causing patients to overeat calorie -rich foods [1]; both can increase weight gain [5]. Similarly, the decreases in physical activity that some psychotropic drugs can produce [6], and the subjective fatigue often described by patients with depression, can diminish calorie expenditure, thereby promoting further weight gain [7]. Environmental and psychological factors often associated with life style of depressed people, like social isolation, chronic underemployment, and low self-esteem, can lead patients to self-medicate with large quantities of “comfort foods” [1].

However, the propensity to obesity among individuals with depression is also sometimes observed in patients who live in apparently-supportive environments and who have not been treated with psychotropic drugs that are recognized as promoting obesity [2]. For example, the overeating and weight gain among populations with LLPDD, a neurohormonal disturbance, seasonal depression with carbohydrate craving [SADS] [8] and anxiety-driven binge eating [9–11], probably have a neurochemical origin and a relationship to the individual’s genetic makeup [12•]. The mechanisms that may be involved are considered below.

## Depression and Related Mood Disorders as Predictors of Future Weight Gain

Compelling evidence that depression can be a risk factor for obesity is provided by the Coronary Artery Risk Development in Young Adults (CARDIA) study [13]. This longitudinal study enrolled over 5000 black and white women and men, aged 18–30, in the mid-l980's, and included follow-up examinations of their mental and physical status at intervals for up to 20 years after enrollment. Using these data, Needham and her co-workers [13] assessed the relationship between the presence of depression at the baseline examination and the development of obesity at the 5, 10, 15, and 20-year examination dates. They found that among white participants, those who were depressed at baseline were significantly more likely to exhibit an increase in body mass index [BMI] than those without initial depressive symptoms, and that depression at baseline was positively correlated with a subsequent increase in waist circumference among both black and white participants. Moreover, the changes in waist circumference were unrelated to gender, race, or such social factors as education or financial status.

Depression in adolescents is also a predictor of obesity [14]. As part of a larger study, the National Longitudinal Study of Adolescent Health, 9000 teenagers in grades 7 through 12 were assessed for depression and obesity at baseline and at follow-up 1 year later [14]. Depressed mood at baseline significantly predicted obesity 1 year later among those who were of normal weight at baseline. Interestingly, baseline obesity did not predict subsequent depression,

A more recent review of the association between depression and obesity further confirmed these findings [2]. A meta-analysis of longitudinal studies examined both the risk of becoming overweight or obese following depression, and conversely, whether obesity increased the risk of developing clinical depression. Bidirectional associations were found: obesity increased the risk of depression by 55% and depression increased the risk of becoming obese by 58%.

## Anxiety, PTSD, and Obesity

Anxiety, with or without depression, may also be a risk factor for obesity and, as several studies have noted [9, 10, 11], it and obesity often go hand in hand. A 2010 literature review [9] revealed a positive association between anxiety disorders and obesity; however, data from longitudinal studies were lacking. Even though there is evidence that anxiety can provoke an increase in appetite, there apparently are no compelling studies showing whether the anxiety syndrome precedes the development of obesity or, more likely, that the two phenomena are concurrent. According to a comprehensive review of eating behavior and emotional state by Silva [15•], anxiety and such other dysphoric mood states as anger, sadness, and stress are often coupled to obesity. An extreme example of this association is seen in *binge eating disorder*, in which excessive quantities of food are ingested over short periods of time by individuals who have severe anxiety or have experienced related negative emotional states [16, 17]. As many as 30% of obese individuals presenting themselves for treatment may have evidence of this overeating disorder. [17].

The emotional stress associated with PTSD can also be a risk factor for the development of obesity. Kubzansky and her colleagues [4] examined weight gain and BMI among a subset of female respondents enrolled in the Nurses’ Health Study II who also met the criteria for PTSD symptoms. Overweight and obesity had previously been recognized as associated with this disorder; however, there was uncertainty as to whether these weight problems first appeared concurrent with the PTSD or the PTSD preceded and might have caused them. The trajectory of weight gain among women in the Nurses ‘Health Study who were diagnosed with PTSD was followed from the onset of symptoms, and weight gain by respondents who had experienced trauma but not PTSD was analyzed separately from those who developed the PTSD syndrome after severe trauma. At every measurement interval, BMI and obesity exhibited significantly greater increases among women who experienced both trauma and PTSD than among women who had trauma but not PTSD.

## Seasonal Affective Disorder (SAD), Premenstrual Syndrome (PMS, LLPDD), and Carbohydrate Craving

Seasonal affective disorder, an atypical form of depression that usually appears in the Northern hemisphere in November–December, the months with the shortest number of daylight hours, is characterized by increased food intake during these months, generally leading to weight gain [18,19,20]. Krauchi and Wirz-Justice [19] compared seasonal changes in food selection between subjects with SAD and normal subjects. They found that those with SAD consumed significantly greater quantities of starchy or sweet carbohydrate-rich foods during months when they experienced SAD than non-affected individuals consumed during the same months. Moreover, individuals with SAD spontaneously decreased their food intake and carbohydrate consumption in months when they were in remission. Direct measurements of calorie and nutrient intake among patients with SAD in November, when they were depressed, and in May, when they were in remission, revealed a selective and significant decrease in calorie and carbohydrate consumption in the spring. [8].

The increased consumption of carbohydrate-rich foods among patients with SAD may be driven by an attempt to use such foods to improve their dysphoric states: patients described an attenuation of their depression after ingestion of carbohydrates, but not after eating protein [8, 20]. A similar pattern of significantly increased carbohydrate and calorie intake in association with cyclic depressions in mood was seen among women experiencing the affective and appetitive symptoms of premenstrual syndrome. When their moods returned to normal during the follicular phase of their menstrual cycle, so too did their carbohydrate intake [21]. In a study on the effects of nutrient consumption on the overeating of carbohydrates among women experiencing premenstrual depression, it was shown that consumption of a carbohydrate-rich meal or snack decreased the depressive symptoms [21]. Measurements of mood, appetite, and cognitive behavior following consumption of a carbohydrate-rich beverage by women with well-defined PMS showed significant improvement in all three sets of behaviors. This effect was not seen following consumption of a beverage rich in protein [22]. Neither test beverage contained fat nor was sweet: In general, non-sweet starches are as likely to be chosen by subjects with depression as sweet ones. The habitual selective consumption of carbohydrate-rich snack foods, daily during the late afternoon or evening, was also observed among a subset of obese, normal individuals who self-identified as carbohydrate cravers. [23]. Following anecdotal reports that carbohydrate snacking followed the onset of mildly-dysphoric moods in the afternoon or evening, changes in mood following consumption of carbohydrate-rich or protein-rich beverages were tested using standardized tests of mood states. These non-depressed subjects also reported significant improvements in mood following carbohydrate but not protein consumption [24].

## Might the Selective Overeating of Carbohydrate-Rich Foods in Association with Dysphoric State Be Predictive of Future Obesity?

The ability of sweet or non-sweet carbohydrate-rich foods to improve mood probably relates to the changes induced by the synthesis and release of the brain neurotransmitter serotonin [25, 26]. The carbohydrates elicit insulin secretion, and the insulin promotes the uptake of most circulating amino acids—especially the large, neutral amino acids [LNAA; principally leucine, isoleucine, valine, tyrosine, phenylalanine, and methionine] into skeletal muscle. However, uptake of tryptophan, serotonin’s precursor amino acid, into skeletal muscle is not substantially increased. Since plasma tryptophan must compete with the other LNAA for uptake into the brain—where some of it can be converted to serotonin—the insulin-induced decrease in plasma LNAA causes more tryptophan to enter the brain and, sequentially, more serotonin to be synthesized and released [8, 25]. This improves the mood [20, 21, 22, 24], much as would happen if patients had increased intrasynaptic serotonin levels by receiving a serotonin-reuptake blocker. It also decreases the concurrent “carbohydrate craving,” and by doing so suppresses the rise in total calorie consumption, especially if the carbohydrate rich foods are also rich in fats.

In normal weight individuals, the consumption of relatively small quantities of carbohydrates (e.g., 25–30 g of carbohydrate in a snack, without protein [26]) induces sufficient insulin secretion to lower plasma levels of these other LNAA thus increasing brain tryptophan levels and serotonin synthesis. Paradoxically, the consumption of dietary proteins—all of which, unlike carbohydrates, do contain tryptophan—fails to elevate tryptophan levels and serotonin synthesis and may actually reduce them [1, 8, 25]. This is because tryptophan is a very minor constituent of dietary proteins (1–1.5%) whereas the LNAA that compete with tryptophan for brain uptake constitute 20–25% of most dietary proteins. Hence, the more protein in a meal or snack (e.g., for example, from Thanksgiving-Day turkey), the *less* brain serotonin is formed and released [25].

When individuals consume a carbohydrate rich, protein-poor snack or meal in association with episodic dysphoric states, the increase in brain serotonin may relieve their symptoms as observed among women with PMS [21, 22], patients with SAD [20], and carbohydrate cravers with or without obesity [24]. Moreover, this overeating of carbohydrates does not necessarily lead to obesity. The cyclic dysphoria occurring in PMS and patients with SAD are of predictable duration and are relieved without further treatment by menstrual cycle changes in blood hormone levels (PMS) or by seasonal changes in length of daily daylight (SAD). The dysphoric moods noted by carbohydrate cravers are time-dependent and may last for only an hour or two. Significant weight gain should not occur if caloric intake does not continue to be elevated when individuals are in remission from SAD and PMS. Carbohydrate cravers can also prevent weight gain if they decrease mealtime calorie intake to compensate for the calories in their snacks. For example, the male respondents in a UK study who became depressed subsequent to consuming large amounts of sugar [27] remained normal in weight.

However, weight gain and subsequent obesity will result if the foods chosen to self-medicate during a dysphoric state happen to be rich in fats as well as carbohydrate. More than 50% of the calories in many pastries, doughnuts, chips, muffins, cookies, ice cream, chocolate, and bagels-with-cream-cheese come from fat. Often portions that are too large are eaten; snacks do not come with instructions to “eat one ounce and wait 30 minutes for your mood to improve.” Unless taught otherwise, the moody, angry, depressed individual with low self-esteem may continue to consume the carbohydrate-and-fat-rich foods until she or he feels better. Thus considerably more than the 25–30 g of carbohydrate needed to promote serotonin synthesis [26] may be ingested. And if the snack happens also to be consumed along with or soon after a protein-rich food, the increase in brain serotonin may be blocked, motivating the eater to consume additional calorie rich snacks an hour or so later.

To break the trajectory from depression to obesity, attention must be paid to whether excessive consumption of carbohydrate-rich foods is being used to modify mood before or during a depressed state and whether this behavior continues even when the depression is in remission. If it does, a food plan can be developed which provides sufficient carbohydrate-rich, protein-poor foods to maintain or increase serotonin levels even when the mood is deteriorating. Unfortunately, patients are sometimes given the opposite advice: they are advised to *minimize* dietary carbohydrate consumption in order to produce weight loss—as in popular diet plans like the Atkins Diet or “Paleo” diets. This strategy is likely to be counterproductive, inasmuch as serotonin synthesis—which depends on brain tryptophan levels [25]—will be slowed, thus exacerbating the carbohydrate craving, depressed mood, and weight gain.

## Conclusion

Depression in adolescence and early adulthood frequently precedes the development of adult obesity. Other common mood disturbances, for example PTSD or anxiety, can similarly promote obesity, concurrently or appearing years later. The mechanism leading from depression to obesity may involve psychotropic drugs that affect appetite or motor activity as a side effect or the consumption of foods that are rich in dietary carbohydrates (but also fats) to enhance brain serotonin synthesis.
